# Supplementary material for: Online search interest in major depressive disorder: Infodemiology study using the most visited search engine in Japan
Source: PLoS One. 2026 May 12;21(5):e0349091. doi: 10.1371/journal.pone.0349091 (PMC13166940; doi:10.1371/journal.pone.0349091)
Supplement: S1 Table — (PDF) [file pone.0349091.s001.pdf]

S1. Table. Codebook for classification of symptoms-related queries based on ICPC-2.

Category	Code	Definition
Pain general/multiple sites	A01	General pain, including non-specific pain.
Fever	A03	Elevated body temperature, including both fever and low-grade fever.
Weakness/tiredness general	A04	General fatigue, weakness, or low energy, including expressions of being unable to move due to lack of energy.
Feeling ill	A05	A general sense of being unwell or malaise.
Limited function/disability NOS	A28	Reduced ability to perform daily activities or functional impairment, including difficulties in work, housework tasks, or other routine activities.
Abdominal pain/cramps general	D01	Abdominal pain, including stomachache.
Nausea	D09	Nausea or feeling sick.
Diarrhoea	D11	Loose stools, frequent bowel movements, or diarrhoea.
Tinnitus, ringing/buzzing ear	H03	Abnormal sounds in the ears without external stimuli.
Palpitations/awareness of heart	K04	Awareness of heartbeat, palpitations, or irregular or rapid heart sensations.
Low back symptom/complaint	L03	Lower back pain or discomfort.
Headache	N01	Headache or head pain.
Abnormal involuntary movements	N08	Problems with movement, coordination, or motor function, including tremor.
Disturbance of smell/taste	N16	Disturbances in smell or taste, including loss, reduction, or alteration of olfactory or gustatory function.
Vertigo/dizziness	N17	Dizziness, vertigo, or sensation of spinning or imbalance.
Feeling anxious/nervous/tense	P01	Anxiety, nervousness, tension, or feelings of unease.

Feeling depressed	P03	Depressed mood or related affective symptoms, including sadness, loss of interest or pleasure, reduced motivation, and crying.
Feeling/behaving irritable/angry	P04	Irritability, anger, or emotional agitation, including expressions of being easily irritated, restless, or aggressive.
Sleep disturbance	P06	Problems of sleep, including insomnia, hypersomnia, irregular sleep patterns, or feeling sleepy.
Memory disturbance	P20	Memory or cognitive disturbances, including forgetfulness, difficulty thinking clearly, reduced concentration, or increased errors.
Psychological symptom/complaint other	P29	Psychological or emotional symptoms not covered by other specific ICPC-2 psychological categories, including abnormal perceptions or thoughts such as hallucinations or delusions.
Suicide/suicide attempt	P77	Suicide or suicidal behavior, including explicit expressions of suicidal ideation or suicide attempts.
Shortness of breath/dyspnoea	R02	Difficulty breathing, shortness of breath, or dyspnoea.
Breathing problem other	R04	Breathing-related complaints other than dyspnoea, including irregular or abnormal breathing patterns such as hyperventilation.
Excessive appetite	T02	Increased appetite, including excessive eating.
Loss of appetite	T03	Decreased appetite or loss of appetite.
Weight gain	T07	Increased body weight.
Weight loss	T08	Decreased body weight.
Limited function/disability (Z)	Z28	Limitations in social functioning, participation, or ability to perform social roles.
