# Supplementary material for: Online search interest in major depressive disorder: Infodemiology study using the most visited search engine in Japan
Source: PLoS One. 2026 May 12;21(5):e0349091. doi: 10.1371/journal.pone.0349091 (PMC13166940; doi:10.1371/journal.pone.0349091)
Supplement: Attachment — Submitted filename: Response-to-Reviewer_Shimizu.docx [file pone.0349091.s003.docx]

March 12, 2026

Dear Dr. Akaishi,

**Title:** Online Search Interest in Major Depressive Disorder: Infodemiology Study Using the Most Visited Search Engine in Japan

**Reference number:** PONE-D-25-64954

Dear Dr. Akaishi,

Thank you for your e-mail of February 3, 2026, regarding our manuscript, “**Online Search Interest in Major Depressive Disorder: Infodemiology Study Using the Most Visited Search Engine in Japan**”, and for the valuable comments of the reviewers. I have attached our revised manuscript, as well as a point-by-point response to the reviewers’ comments.

We hope that the revised manuscript contains suitable responses to the comments, and we think that it has been significantly improved over the previous submission. We trust that our manuscript is now suitable for publication in PLOS ONE.

Thank you in advance for your kind consideration of our work.

Sincerely yours,

Kosuke Ishizuka, MD, PhD

Department of General Medicine, Yokohama City University School of Medicine

3-9 Fukuura, Kanazawa-ku, Yokohama-city, Kanagawa pref. Japan

Tel. +81-45-787-2800

Fax. +81-45-350-2728

E-Mail: e103007c@yokohama-cu.ac.jp

**RESPONSES TO REVIEWER 1:**

We sincerely thank the reviewer for insightful comments that have helped us to improve our paper. We believe that the revised version adequately addresses all concerns raised, and we think that it has been significantly improved over the previous submission. We trust that our manuscript is now suitable for publication in PLOS ONE.

## **NOTE**

We highlighted changes of significant issues in yellow (please see “Revised Manuscript with Track Changes”).

**Comments:**

This descriptive study uses Yahoo! JAPAN search queries (2022–2024) to characterize public interest in major depressive disorder (“Utsu-byou”), focusing on symptoms co-searched with MDD and classifying them via ICPC-2 with mapping to DSM-5-TR criteria. The taxonomy-based classification is a strength and could be useful for infodemiology and health communication.
However, I have some concerns that should be addressed before publication.

**Response:**

Thank you for your general comments. In accordance with your suggestions, we have revised the manuscript. We have addressed each of the points you raised.

**Major comments:**

1. (L66) "The level of interest in MDD among members of the general population remains largely unexplored and research on public interest in MDD through online search behavior is limited."
The current background does not sufficiently review prior work on using online search behavior to characterize public interest in mental health conditions. Please strengthen the literature review and clarify the specific gap this study addresses.

**Response:**

We agree with your assessment. Along with your suggestion, we have revised the Introduction to include a more detailed review of prior research and to clarify the novelty of the present study. We also modified the Abstract accordingly.

**Changes:**

- Previous studies on public interest in MDD based on online search trends have addressed temporal changes and regional differences, whereas analyses focusing on the content of search queries remain limited. (Abstract; lines 32 to 34.)
- More specifically, previous research has largely been confined to examining the association between depression risk and indicators such as temporal fluctuations and geographic variation in search volume using Google Trends [10-12]. In contrast, studies that directly examine the substantive content of search queries remain scarce in part because Google Trends provides limited access to detailed information on the specific content of search queries. (Introduction; lines 73 to 78.)

2. (L68) "Therefore, this study aimed to characterize public interest in MDD in Japan using online search data and to describe the temporal trends in online searches related to MDD in Japan by year during the period 2022 to 2024."
Please provide a clearer rationale for focusing on Japan and the 2022–2024 period. As written, the choice of country and time window feels arbitrary. Briefly explain whether this is driven by data availability, contextual factors, policy/health system considerations, or specific events that make this period particularly relevant.

**Response:**

We thank the reviewer for this valuable comment. We agree that the rationale for selecting the country and study period was insufficiently explained. We have added an explanation in the Introduction regarding the social background supporting our focus on Japan. In addition, we have clarified in the Methods section the reasons for selecting the three-year period, including data availability and the temporal stability of the data.

**Changes:**

- In particular, Japanese individuals are often described as being reluctant to discuss mental illness with others [6]. In such a sociocultural context, the anonymity of online search may render it a preferable means of seeking information. (Introduction; lines 65 to 68.)
- The period from 2022 to 2024 was selected based on considerations of data availability and stability. (Methods; lines 87 to 88.)

3. (L75) "the term “Utsu-byou” (the Japanese term for MDD)"
Japanese queries have common orthographic variants (e.g., うつ病 vs 鬱病) and may also be used colloquially without implying a clinical diagnosis. Please clarify whether variants were tested and/or included, and consider sensitivity analyses comparing results across key variants. In addition, because “Utsu-byou” searches may include substantial noise, please discuss how this could affect the findings and, if feasible, provide the exact Japanese search terms used (e.g., in a supplement).

**Response:**

As you have indicated, we have clarified in the Methods that utsu-byo refers to the Japanese term “うつ病,” In addition, we have added an explanation in the Discussion section regarding orthographic variations specific to the Japanese language and the potential bias arising from such variations as a study limitation.

**Changes:**

- To investigate trends in online searches related to MDD, we obtained the number of search queries containing the term “うつ病” (Utsu-byo, Depression; Major Depressive Disorder) from Yahoo! JAPAN for the period from January 2022 to December 2024. (Methods; lines 85 to 87.)
- Eighth, there is a possibility of bias due to orthographic variants. In Japanese, “Utsu” can be written in both hiragana and kanji, resulting in two forms, “うつ” and “鬱”, and in this study we adopted the form “うつ”. The kanji character “鬱” was added to the list of commonly used kanji in 2010 [36]. In practice, however, the form “うつ病” is more widely used in everyday contexts and is more familiar to the general public. The Japanese version of the DSM-5 and DSM-5-TR also adopts “うつ病”, indicating that it is the standard term in medical settings [13]. For these reasons, we selected “うつ病” to ensure terminological and clinical consistency. Variations in orthography may be associated with differences in demographic characteristics, such as gender or age group. However, because the data obtained in this study included only queries containing “うつ病”, potential differences related to alternative forms were not examined and should be addressed in future research. (Discussion; lines 303 to 313.)

4. (L76) "Yahoo! JAPAN is the most frequently used search engine in Japan,"
This statement should be carefully verified and appropriately framed. Metrics such as site visits to yahoo.co.jp (a large portal including news, weather, mail, etc.) are not equivalent to search engine market share. If the intent is to justify platform representativeness, please use a metric directly relevant to search usage (e.g., search engine market share) and revise the wording accordingly. Please cite an appropriate source.
Ideally, provide a supplementary table listing the symptom terms (and representative raw queries) with final ICPC-2 labels and DSM alignment.

**Response:**

We appreciate the reviewer’s suggestion. We changed the metric for Yahoo! usage from the number of visits to yahoo.co.jp to its search engine market share and added information on highlighting Yahoo!’s advantages. We note, however, that the data and analyses obtained through DS.INSIGHT are subject to contractual restrictions and cannot be shared with third parties, and therefore cannot be provided in this study.

**Changes:**

- Yahoo! JAPAN has maintained the second-largest market share among search engines in Japan for many years, following Google [14]. Yahoo! provides data on the top 500 queries containing specific terms, along with information on the gender and age group of the users who performed these searches. On the other hand, Google Trends, which retrieves data from Google, provides only the regional interest trends for each query, the top 50 related queries, and the top 50 queries with rising search volume. In addition, web audience measurement data indicate that Yahoo! JAPAN continues to be widely used in Japan, with approximately 67.7% of the population using the service at least once per month in 2024, compared with 68.8% for Google during the same period [15,16]. (Methods; lines 94 to 102.)

5. (L88) "Search queries used in the analysis"
⇒Without a codebook (decision rules) and examples, readers cannot evaluate how raw queries were mapped to symptom terms and then to ICPC-2 categories—especially for slang, spelling variants, multi-term queries, and ambiguous expressions.
Moreover, because two coders independently classified terms with third-party adjudication, please report inter-rater reliability (e.g., Cohen’s kappa and/or percent agreement) and the proportion of disagreements requiring adjudication.

**Response:**

Thank you for this helpful comment. To improve transparency of the classification process, we have added additional methodological details in the revised manuscript, including information on inter-rater reliability (Cohen’s kappa) and examples illustrating how search queries with spelling variations or colloquial expressions were categorized.

**Changes:**

- Representative classification rules were applied; for example, both “Depression can’t sleep” and “Depression just sleeping all day” were classified under “Sleep disturbance”, allowing for consistent categorization across slang and variant expressions. As a representative example, Cohen’s kappa coefficient was calculated for 93 symptom queries from 2024, yielding κ = 0.751, indicating substantial inter-rater agreement and sufficient reproducibility of the classification. (Methods; lines 136 to 141.)

6. (L173) "Table 3. Top 20 search terms and symptoms in online searches related to major depressive disorder by year (2022–2024)."
Please also report proportions to enable valid comparisons across years.

**Response:**

We have reflected this comment by adding the proportion of each query’s search volume relative to the searches for “Depression” for each year in Table 3.

**Changes:**

- Table 3

7. (L221) "Given that the diagnostic criteria of the DSM-5-TR are based on a statistical classification of clinically observed symptoms and signs, individuals searching online for
symptoms of MDD may include patients diagnosed with MDD."
⇒Search volume reflects information-seeking behavior rather than clinical status; therefore, findings should be interpreted as public interest in symptom-related concepts linked to “Utsu-byou,” not as evidence of symptom prevalence or diagnosis. Please revise the wording accordingly to avoid potential misunderstanding.

**Response:**

Thank you for your important assessment. Although we have noted in the limitation that caution is needed regarding the interpretation of searchers, to avoid the potential misunderstanding that all searchers are patients with depression, we have removed the cited sentence and the following sentence, and revised the Discussion accordingly.

**Changes:**

- This finding suggests that public awareness of depressive symptoms may be partially aligned with clinical diagnostic concepts. (Discussion; lines 249 to 251.)
- [Deleted] ~~Given that the diagnostic criteria of the DSM-5-TR are based on a statistical classification of clinically observed symptoms and signs, individuals searching online for symptoms of MDD may include patients diagnosed with MDD. For example, in cases in which patients have already been diagnosed with MDD, they may be skeptical about the doctor’s diagnosis and explanation or feel dissatisfaction and anxiety when the diagnosis is perceived as inappropriate or when treatment fails to alleviate symptoms, leading patients to seek to confirm the diagnosis or obtain further information about MDD symptoms online. Moreover, in cases in which MDD has not been formally diagnosed by a health professional, some individuals may suspect that they or an associate have MDD based on the symptoms and conduct online searches to obtain further information. In addition, in cases of severe MDD, in which psychomotor retardation and loss of motivation are common, those around affected individuals may be more likely than the affected individuals themselves to carry out searches on MDD symptoms.~~

**Minor comments:**

1. (L83) "https://datasolution.yahoo-net.jp/view/knowledgebase/641."
If this resource requires a login or is not publicly accessible, it may limit reproducibility for readers. Please provide a publicly accessible reference, or alternatively describe the relevant details in the manuscript or a supplementary appendix.

**Response:**

Along with your comments, we have updated the URL to a publicly accessible one.

**Changes:**

- The user manual is available at https://datasolution.yahoo-net.jp/view/knowledgebase/2205. (Methods; lines 108 to 109.)

## **Others**

We also indicated other minor corrections in yellow markers (please see “Revised Manuscript with Track Changes”).

We thank the reviewer for such pertinent comments. We believe that the revised manuscript adequately addresses the reviewers’ comments and has been substantially improved compared to the previous version. We hope that it is now suitable for publication in PLOS ONE.

**RESPONSES TO REVIEWER 2:**

We sincerely thank the reviewer for their insightful comments, which have greatly contributed to improving our manuscript. We believe that the revised version addresses all the concerns raised and represents a significant improvement over the previous submission. We hope that it is now suitable for publication in PLOS ONE.

## **NOTE**

We highlighted changes of significant issues in yellow (please see “Revised Manuscript with Track Changes”).

**Comments:**

The manuscript “Online Search Interest in Major Depressive Disorder: Infodemiology Study Using the Most Visited Search Engine in Japan” sets out to characterize public interest in Major Depressive Disorder (MDD) in Japan using Yahoo! search trends from 2022 to 2024. The authors compare the most frequent terms co-searched with queries for major depressive disorder (MDD) to official classifications of MDD symptoms. Internet search data offers promising opportunities to measure public interest and public health, and we still need a clearer understanding of when and how researchers can meaningfully apply these data. I applaud the authors for advancing our understanding in this direction. However, I have two major concerns with the paper at hand regarding (1) the study goal and relevance, (2) the study’s empirical approach, and (3) the presentation of results. I elaborate on these points, along with several more minor comments, below. I hope that my comments are constructive and helpful to the authors in further strengthening their research.

**Response:**

Thank you for your general comments. In accordance with your suggestions, we have revised the manuscript. We have addressed each of the points you raised.

**Major comments:**

(1) Study goal and relevance: The authors should state the study’s research question, objectives, and scientific and societal relevance more clearly from the outset. In the introduction, the authors note that major depressive disorder (MDD) is frequently underdiagnosed or diagnosed with delay and state that the study aims “to characterize public interest in MDD” (p. 3, line 68). However, it remains unclear how the goal of characterizing public interest in MDD relates to the societal problem of delayed MDD diagnoses. It is also not specified in which contexts and for whom such a characterization would be useful and what the search interest in MDD over time would tell us.
The conclusion further states that “focusing on these symptoms may enable primary care physicians to proactively identify cases of MDD” (p. 15, lines 308 to 209). Given that the classification of Yahoo! searches is based on the official classification of MDD symptoms, it remains unclear, however, whether this approach offers information beyond what medical practitioners already rely on clinical practice.
Throughout the manuscript, it is confusing whether the study’s goal is to assess whether online search data could function as an early detection system for MDD by predicting official diagnosis rates, or whether it seeks to improve diagnostic understanding by identifying the most frequently searched MDD symptoms. Clarifying the overarching objective of the study from the outset would substantially strengthen the manuscript.

**Response:**

We are grateful for this important point. We have clarified the objective of this study and added explanations in the Introduction and Abstract to better align it with the research questions. In addition, we have expanded the Discussion and Conclusion to more clearly articulate the significance of this study. Accordingly, the Abstract has also been revised.

**Changes:**

- This study aimed to describe online search trends on MDD in Japan and enhance understanding of diagnostic practices by examining how depression is perceived within society. (Abstract; line 34 to 36.)
- Public perceptions online of depressive symptoms may demonstrate a certain degree of consistency with clinical diagnostic concepts. Focusing on symptoms that attracted high attention online may facilitate more proactive detection of MDD in primary care settings. (Abstract; line 48 to 51.)
- Additionally, we sought to clarify social perceptions of depression to deepen understanding of its diagnosis. (Introduction; line 80 to 81.)
- The findings of this study suggest that the general public may search for symptoms related to themselves or those around them in connection with depression, and that the symptoms of greatest interest are consistent with the diagnostic criteria outlined in the DSM-5-TR. This alignment illuminates social perceptions of depression as reflected in online search behavior. Future research should investigate the relationship between depression or help-seeking behaviors and search activity. Building on these investigations, focusing on symptoms that attracted substantial online attention may facilitate more proactive detection of MDD in primary care settings. (Discussion; line 271 to 278.)
- These findings illuminate social perceptions of depression and its symptoms as reflected in online search behavior. Further research is needed to determine the extent to which such behavior reflects clinical condition. This line of research may help primary care physicians to proactively identify patients with MDD by focusing on these commonly searched symptoms. (Conclusion; line 322 to 326.)

The discussion should then be more tightly aligned with this clarified objective. Currently, the discussion contains statements that do not reflect any of the goals hinted at in the introduction. For instance, the authors state that “promoting acceptance of MDD among patients and those around them is essential” (p. 11, lines 206 to 208), suggesting an additional goal related to stigma reduction. However, the manuscript does not demonstrate how the presented findings inform or advance this objective. Similarly, the statement that “when disseminating information about diseases, ensuring the accuracy of the content is important” (p.12, lines 236 to 238) is connected to the empirical analysis, as the study does not examine the content of information presented to users following their Yahoo! searches. Overall, the authors must specify the broader goal of their research, focus their abstract, introduction, and discussion on this goal, and streamline their statements in these chapters accordingly.

**Response:**

We agree with the reviewer’s comment. After careful discussion, we concluded that this content was outside the scope of our study and have therefore removed it.

**Changes:**

- [Deleted] ~~Some patients who visit health professionals may have already searched for symptoms of MDD prior to their consultation. Therefore, in clinical practice, careful evaluation during the diagnostic work-up and efforts to foster a good doctor-patient relationship may be beneficial. In addition, those who search for “MDD symptoms” may include patients and those around them who question their MDD diagnosis. MDD is often associated with negative perceptions, and such stigma can hinder the acceptance of a MDD diagnosis [16]. Thus, to achieve early diagnosis and timely therapeutic intervention, and promoting acceptance of MDD among patients and those around them is essential.~~
- [Deleted] ~~Internet searches are one of the most convenient means for the general public to obtain information about diseases. As noted above, they have been suggested to influence perception of the disease and the formation of the sick role [14,15]. Therefore, when disseminating information about diseases, ensuring the accuracy of the content is important. In particular, individuals may feel dissatisfied and anxious when diagnosed with MDD.~~

Finally, the implications of the study for existing and future research are not sufficiently stated, leaving it unclear how researchers might build on the reported findings. The abstract currently notes that “research on public interest in MDD based on online search trends is limited” (p. 2, lines 33 to 34) but does not discuss whether prior studies have attempted to predict MDD using digital trace data or how successful such efforts have been. The abstract and introduction should more clearly situate the study and its contribution within the existing literature, while the discussion should explicitly outline the study’s implications for future research.

**Response:**

We thank the reviewer for this valuable comment. We agree that this aspect was not sufficiently explained. We have clarified prior infodemiology research on depression and the position of the present study in the Introduction, and revised the Abstract accordingly. We also added a description of the study’s potential future applications in the Discussion.

**Changes:**

- Previous studies on public interest in MDD based on online search trends have addressed temporal changes and regional differences, whereas analyses focusing on the content of search queries remain limited. (Abstract; lines 32 to 34.)
- More specifically, previous research has largely been confined to examining the association between depression risk and indicators such as temporal fluctuations and geographic variation in search volume using Google Trends [10-12]. In contrast, studies that directly examine the substantive content of search queries remain scarce in part because Google Trends provides limited access to detailed information on the specific content of search queries. (Introduction; lines 73 to 78.)
- The findings of this study suggest that the general public may search for symptoms related to themselves or those around them in connection with depression, and that the symptoms of greatest interest are consistent with the diagnostic criteria outlined in the DSM-5-TR. This alignment illuminates social perceptions of depression as reflected in online search behavior. Future research should investigate the relationship between depression or help-seeking behaviors and search activity. Building on these investigations, focusing on symptoms that attracted substantial online attention may facilitate more proactive detection of MDD in primary care settings. (Discussion; lines 271 to 278.)

(2) Empirical approach: For other Internet search data sources such as Google Trends, previous research has shown that the selection of appropriate search terms poses substantial challenges for establishing internal (measurement) validity (e.g., Mellon 2013, 2014; Hölzl et al. 2025). Similar concerns are likely to apply to the use of Yahoo! search data. Individuals interested in or concerned about MDD may not necessarily search for the term “Utsu-byou,” but instead use other terms. The authors thus need to explain their search term selection of “Utsu-byou” and make a strong case for why people would search for exactly this term when concerned about MDD. In this context, it would be helpful to clarify whether “Utsu-byou” is a commonly used term in Japan.

**Response:**

We sincerely thank the reviewer for the thoughtful comment and for providing relevant references. As orthographic variations may occur in the term “Utsu-byou” due to characteristics of the Japanese language, we have added a statement addressing this issue as part of the Discussion. In addition, we have included an explanation of the translation of “Utsu-byou” in the Methods section.

**Changes:**

- In this study, “Depression” was used to represent queries searched by the general public, whereas the clinical concept was translated as “Major Depressive Disorder”. In Japanese, the term “うつ病” can refer both to depression in a general sense and to MDD as defined in the DSM‑5‑TR [13]. Therefore, it is not possible to determine which meaning a search query intends, and the term was translated in this way. (Methods; lines 88 to 93.)
- Eighth, there is a possibility of bias due to orthographic variants. In Japanese, “Utsu” can be written in both hiragana and kanji, resulting in two forms, “うつ” and “鬱”, and in this study we adopted the form “うつ”. The kanji character “鬱” was added to the list of commonly used kanji in 2010 [36]. In practice, however, the form “うつ病” is more widely used in everyday contexts and is more familiar to the general public. The Japanese version of the DSM-5 and DSM-5-TR also adopts “うつ病”, indicating that it is the standard term in medical settings [13]. For these reasons, we selected “うつ病” to ensure terminological and clinical consistency. Variations in orthography may be associated with differences in demographic characteristics, such as gender or age group. However, because the data obtained in this study included only queries containing “うつ病”, potential differences related to alternative forms were not examined and should be addressed in future research. (Discussion; lines 303 to 313.)

The classification of search terms co-occurring with MDD-related queries into MDD symptoms represents a very valuable component of the study’s empirical approach. However, the description of the coding procedure would benefit from further clarification. Specifically, the authors note that “an additional evaluation by a third researcher (KI) was performed to minimize observer bias” (p. 5, lines 96–97), but it is not specified how this evaluation was conducted. Typically, reducing observer bias would involve independent coding of a subset of the data by multiple researchers, the calculation of interrater reliability, and the joint resolution of any discrepancies in the coding. The authors should clarify whether multiple coders independently classified a subset of search terms and, if so, report the corresponding interrater reliability. If the third researcher’s role differed from this standard procedure, further explanation is needed.

**Response:**

Thank you for this helpful comment. To improve the transparency of the classification process, we have added additional methodological details in the revised manuscript. We also included information on inter-rater reliability (Cohen’s kappa) and provided examples illustrating how search queries with spelling variations or colloquial expressions were categorized.

**Changes:**

- For the symptom classification, each term was independently evaluated by two researchers (RS and KK), and the results were subsequently compared. Then, an additional evaluation by a third researcher (KI) was performed to minimize observer bias. Representative classification rules were applied; for example, both “Depression can’t sleep” and “Depression just sleeping all day” were classified under “Sleep disturbance”, allowing for consistent categorization across slang and variant expressions. As a representative example, Cohen’s kappa coefficient was calculated for 93 symptom queries from 2024, yielding κ = 0.751, indicating substantial inter-rater agreement and sufficient reproducibility of the classification. (Methods; lines 133 to 141.)

The manuscript would also benefit from a clearer justification of the case selection. The authors should explain why Japan constitutes a particularly relevant or informative context for their research purposes. Similarly, they should discuss why they selected the two-year analysis period from 2022 to 2024. The authors need to make a case for why the search trends for these two years is particularly interesting for their research purposes.

**Response:**

We thank the reviewer for this valuable comment. We agree that the rationale for selecting the country and study period was insufficiently explained. We have added an explanation in the Introduction regarding the social background supporting our focus on Japan. In addition, we have clarified in the Methods section the reasons for selecting the three-year period, including data availability and the temporal stability of the data.

**Changes:**

- In particular, Japanese individuals are often described as being reluctant to discuss mental illness with others [6]. In such a sociocultural context, the anonymity of online search may render it a preferable means of seeking information. (Introduction; lines 65 to 68.)
- The period from 2022 to 2024 was selected based on considerations of data availability and stability. (Methods; lines 87 to 88.)

Finally, the authors should address the quality of the Yahoo! search data. Providing information on data coverage, reliability, and any known limitations of Yahoo!’s search data would strengthen confidence in the empirical findings.

**Response:**

Along with your recommendation, we have replaced the URL for the DS.INSIGHT user manual with a publicly accessible link and added a reference URL describing the data definitions.

**Changes:**

- The user manual is available at https://datasolution.yahoo-net.jp/view/knowledgebase/2205. Data definitions can be accessed from https://datasolution.yahoo-net.jp/view/knowledgebase/post/2596. (Methods; lines 108 to 111.)

(3) Presentation of results: In the description of the results, the authors currently simply list the frequencies of search queries (e.g., p. 7, line 140, to p. 9, line 175). The authors should focus more on telling a story that aligns with the research’s goal on how these searches relate to the detection of MDDs or the improvement of diagnostics. If the research goal lies in prediction of MDD instead of improving diagnostics, the authors need to add predictions of official statistics of MDD from the Yahoo! searches. More generally, the description and discussion of findings feel quite lengthy and should be condensed.

**Response:**

Thank you for your important comment. After careful consideration, we revised the Results by retaining only the parts relevant to the Discussion and removing the others, and we have provided additional appropriate explanations. We also revised the Discussion by deleting content that was not aligned with the objectives of this study and refining the relevant descriptions accordingly.

**Changes:**

- [Deleted] ~~Search terms ranked fourth or lower varied by year. Specifically, in 2022, the fourth most common query was “how to overcome MDD” (n = 82,500) and the fifth most common query was “bipolar disorder test” (n = 45,500). In 2023, the fourth most common query was “difference between MDD and adjustment disorder” (n = 54,500) and the fifth most common query was “MDD how to help” (n = 48,600). In 2024, the fourth most common query was “difference between MDD and adjustment disorder” (n = 85,900) and the fifth most common query was “how to overcome MDD”(n = 51,500) (Table 2).~~
- The most frequently searched queries related to depressive symptoms are shown in Table 3. Symptoms corresponding to the DSM-5-TR diagnostic criteria for MDD are indicated by underlining. “Sleep disturbance” was the most frequently searched symptom in all years. “Feeling depressed”, “Feeling/behaving irritable/angry”, “Weakness/tiredness general”, “Memory disturbance”, “Suicide/suicide attempt” were consistency ranked within the top 10 across all years. All of these symptoms were consistent with the diagnostic criteria of the DSM-5-TR for MDD. In addition, among the symptoms not included in the DSM-5-TR diagnostic criteria, “Headache” ranked first in 2023 and 2024, excluding the residual category. (Results; lines 207 to 214.)
- [Deleted] ~~In 2022, the most frequently searched symptom of MDD was “sleep disturbance” (n = 36,710), followed by “feeling depressed” (n = 10,150), “feeling/behaving irritable/angry” (n = 10,120), “memory disturbance” (n = 9,290), “suicide/suicide attempt” (n = 8,680), “limited function/disability” (n = 7,650), “general weakness/tiredness” (n = 6,540), “feeling anxious/nervous/tense” (n = 5,280), “psychological symptom/complt other” (n = 4,980), and “headache” (n = 4,800) (Table 3).In 2023, “sleep disturbance” ranked first (n = 24,050), followed by “feeling/behaving irritable/angry” (n = 13,570), “feeling depressed” (n = 11,960), “limited function/disability” (n = 11,360), “memory disturbance” (n = 7,480), “general weakness/tiredness” (n = 7,390), “suicide/suicide attempt” (n = 5,500), “psychological symptom/complt other” (n= 5,160), “loss of appetite” (n = 4,300), and “headache” (n = 4,190) (Table 3).In 2024, “sleep disturbance” remained the most common search query (n = 21,380), followed by “feeling depressed” (n = 12,280), “limited function/disability” (n = 11,860), “feeling/behaving irritable/angry” (n = 10,600), “general weakness/tiredness” (n = 7,650), “psychological symptom/complt other” (n = 5,900), “memory disturbance” (n = 5,710), “suicide/suicide attempt” (n = 4,970), “headache” (n = 4,400), and “loss of appetite” (n = 4,200) (Table 3).~~
- [Deleted] ~~Some patients who visit health professionals may have already searched for symptoms of MDD prior to their consultation. Therefore, in clinical practice, careful evaluation during the diagnostic work-up and efforts to foster a good doctor-patient relationship may be beneficial. In addition, those who search for “MDD symptoms” may include patients and those around them who question their MDD diagnosis. MDD is often associated with negative perceptions, and such stigma can hinder the acceptance of a MDD diagnosis [16]. Thus, to achieve early diagnosis and timely therapeutic intervention, and promoting acceptance of MDD among patients and those around them is essential.~~
- [Deleted] ~~Given that the diagnostic criteria of the DSM-5-TR are based on a statistical classification of clinically observed symptoms and signs, individuals searching online for symptoms of MDD may include patients diagnosed with MDD. For example, in cases in which patients have already been diagnosed with MDD, they may be skeptical about the doctor’s diagnosis and explanation or feel dissatisfaction and anxiety when the diagnosis is perceived as inappropriate or when treatment fails to alleviate symptoms, leading patients to seek to confirm the diagnosis or obtain further information about MDD symptoms online. Moreover, in cases in which MDD has not been formally diagnosed by a health professional, some individuals may suspect that they or an associate have MDD based on the symptoms and conduct online searches to obtain further information. In addition, in cases of severe MDD, in which psychomotor retardation and loss of motivation are common, those around affected individuals may be more likely than the affected individuals themselves to carry out searches on MDD symptoms.~~
  ~~Internet searches are one of the most convenient means for the general public to obtain information about diseases. As noted above, they have been suggested to influence perception of the disease and the formation of the sick role [14,15]. Therefore, when disseminating information about diseases, ensuring the accuracy of the content is important. In particular, individuals may feel dissatisfied and anxious when diagnosed with MDD.~~

Table 1 and 2 would also very much benefit from a clarification on the absolute numbers of search volume. For instance, it is not explained what the numbers of the search volume in the column “Internet user” mean and how they have been calculated. The table note should explain the metric. In general, the table notes should be more informative so that readers are able to understand the text without the table and vice versa.

**Response:**

We thank the reviewer for valuable comments. In Table 1, “Internet user” represents population counts. To avoid confusion, the term “search volume” was changed to “volume.” In Tables 1, 2, and 3, we also added more detailed information, including explanations of the meaning of each value, the calculation methods, and other relevant details.

**Changes:**

- ^a^The internet user population was defined as the number of the internet users throughout Japan, calculated from the population of Japan by sex and age group and the corresponding the internet usage rates based on the “Communication Usage Trend Survey” and the “Population Estimates” conducted by the Ministry of Internal Affairs and Communications.
  ^b^“Search term ‘depression’” indicates the search volume for the query “depression” and the proportion of searches by each group.
  ^c^Proportions were calculated using the total number of internet users and the total search volume for the query “Depression” in each year as 100%, respectively. (Results; lines 185 to 192.)
- The search volume for each query is shown in parentheses. The search queries were originally collected in Japanese and translated into English by the author. (Results; lines 204 to 205.)
- The underlined terms indicate symptoms of major depressive disorder listed in the Diagnostic and Statistical Manual of Mental Disorders, Fifth Edition, Text Revision (DSM-5-TR). The search volume for each query is shown in parentheses. The search queries were originally collected in Japanese and translated into English by the author. (Results; lines 219 to 222.)

**Minor comments:**

Abstract: In the abstract, the results are described in too much detail, relatively little attention is given to the broader implications of the findings. It would be helpful for readers to grasp from the abstract what the study’s implications are.

**Response:**

We sincerely thank the reviewer for this helpful comment. As the results was overly long, we have shortened it and added a statement regarding the significance of this study in the conclusion.

**Changes:**

- Specifically, sleep disturbance, which is also frequently observed in clinical practice, was the most frequently co-searched symptom. Public perceptions online of depressive symptoms may demonstrate a certain degree of consistency with clinical diagnostic concepts. Focusing on symptoms that attracted high attention online may facilitate more proactive detection of MDD in primary care settings. (Abstract; lines 47 to 51.)

p. 3, 51: The term “Major Depressive Disorder” should be defined somewhere. Is MDD the clinical term for what lay people understand under the term depression?

**Response:**

You have raised an important question. In Japanese, both “depression” and “MDD” are expressed as “うつ病.” In light of this fact and the reviewer’s comment, we have replaced “MDD” which is used as queries with “depression” throughout the manuscript.

**Changes:**

- In this study, “Depression” was used to represent queries searched by the general public, whereas the clinical concept was translated as “Major Depressive Disorder”. In Japanese, the term “うつ病” can refer both to depression in a general sense and to MDD as defined in the DSM‑5‑TR [13]. Therefore, it is not possible to determine which meaning a search query intends, and the term was translated in this way. (Method; lines 88 to 93.)

p. 3, 57-59: When discussing this study, it should be stated when the study was conducted and for which regions the results apply.

**Response:**

As you have indicated, we have added information regarding the study period and the geographic area covered by the research.

**Changes:**

- A previous study reported in 2017 found that approximately 60% of individuals who met the DSM-IV criteria for MDD in the past 12 months had not received treatment in 12 high-income countries [4]. (Introduction; lines 60 to 63.)

p. 4, 76ff.: The authors cite a source showing the number of Yahoo! users compared to Google users in Japan in 2021. Has the proportion of Yahoo! users to Google users changed within the last five years? As Google users have also been a substantially large group in 2021, the authors should discuss in the discussion chapter how Yahoo! and Google users differ from each other, i.e., whether there are any systematic differences between the two groups.

**Response:**

We thank the reviewer for this helpful comment. We have updated the data to the most recent available version and added an explanation regarding the differences between Yahoo! users and Google users.

**Changes:**

- Yahoo! JAPAN has maintained the second-largest market share among search engines in Japan for many years, following Google [14]. (Methods; lines 94 to 95.)
- In Japan, individuals under 30 years old use Google almost twice as often as Yahoo! JAPAN, while users aged 40 and above tend to favor Yahoo! JAPAN over Google [17]. (Methods; lines 102 to 104.)

p. 4, 82: It should already be added here that Yahoo! gets the information on age and gender from the users’ profiles

**Response:**

The manuscript has been revised as suggested.

**Changes:**

- Yahoo! JAPAN determines users’ age and gender based on the information registered in their user accounts. (Methods; lines 110 to 111.)

p. 4, 83: As only registered users have access to the user manual, it would be very helpful if the authors would provide some openly accessible documentation of Yahoo! JAPAN DS.INSIGHT so that readers can better inform themselves about the tool.

**Response:**

Along with your comments, we have updated the URL to a publicly accessible one.

**Changes:**

- The user manual is available at https://datasolution.yahoo-net.jp/view/knowledgebase/2205. (Methods; lines 108 to 109.)

p. 4, 84ff.: The authors should explain for which research purposes they additionally collected survey data. They also need provide a citation for the survey and provide more information on the survey, such as the field period and number of respondents. Similarly, they need to outline what the “Population Estimates” by the Ministry of Internal Affairs and Communications are, why they are needed for their research purposes, and correctly cite this data source, as well.

**Response:**

We acknowledge this issue and have addressed it as follows. We have clarified the purpose and content of “Communication Usage Trend Survey” and “Population Estimates,” and have revised the corresponding references appropriately.

**Changes:**

- The internet user volume was defined as the number of internet users in Japan. To define the attributes of the population underlying the search data, we calculated the volume of the internet users in Japan by sex and age group based on the proportion of the internet use from the “Communication Usage Trend Survey” and population data from the “Population Estimates” conducted by the Ministry of Internal Affairs and Communications [18-21]. The “Communications Usage Trend Survey” is a statistical survey conducted to assess trends in telecommunications usage, included 39,577 respondents in 2022, 34,196 in 2023, and 37,058 in 2024 [18-20]. The “Population Estimates” provide population estimates by sex and age group based on the national census and other demographic data reflecting population changes [21]. In this study, population data as of October 1 of each year were used. (Methods; lines 115 to 124.)

p. 4, 90: The authors state that they compared the search volume of queries that also include symptoms in the query. To get a better overall picture of the content of MDD queries, it would be helpful if the authors could also provide what the other search terms searched along with MDD were that are not symptoms.

**Response:**

Thank you for your suggestion. We note, however, that the data and analyses obtained through DS.INSIGHT are subject to contractual restrictions and cannot be shared with third parties without reasonable request, and therefore cannot be provided in this study outside of Table1. This explanation has been added to the Method Section.

**Changes:**

Access to the data used in this research is restricted in accordance with the policies of Yahoo! JAPAN Corporation. The analyses were conducted in compliance with the terms and conditions governing the use of Yahoo! JAPAN DS.INSIGHT. (Methods; lines 111 to 114.)

p. 5, 105: In the formula for the calculation of the z-scores, does the average search volume of query A relate to the average across all months in the data collection period? If yes, this should be clearly denoted in the formula.

**Response:**

The average search volume of query A, as used in the z-score calculation, is the mean of all monthly values within the data collection period. We have explicitly stated this in the revised manuscript.

**Changes:**

- $\bar{Search volume of query A}$ represents the mean search volume of query A across all months in the study period. (Methods; lines 155 to 156.)

p. 6, 125f.: In “50–51% of internet users were male, and the user population was comparatively evenly distributed across the different age groups”, it is unclear whether the numbers reported relate to the survey data, the “Population Estimates” by the Ministry of Internal Affairs and Communications, or the Yahoo! users only.

**Response:**

We have added an explanation of the values presented in Table 1.

**Changes:**

- Table 1 presents the estimated population of the internet users in Japan, derived from the “Communication Usage Trend Survey” and “Population Estimates”, alongside the search volume for the query “Depression,” broken down by sex and age group [18-21]. (Results; lines 173 to 175.)

p. 7, 135f.: The definition of the internet user population from the table note should be included in the description of the data sources as well (p. 4, lines 84-86).

**Response:**

We have added a detailed explanation in the Methods section regarding the definition of Internet users and how they were calculated.

**Changes:**

- The internet user volume was defined as the number of internet users in Japan. To define the attributes of the population underlying the search data, we calculated the volume of the internet users in Japan by sex and age group based on the proportion of the internet use from the “Communication Usage Trend Survey” and population data from the “Population Estimates” conducted by the Ministry of Internal Affairs and Communications [18-21]. (Methods; lines 115 to 119.)

p. 8, 168: There potentially is a spelling error in “psychological symptom/complt other”.

**Response:**

We appreciate the reviewer’s careful reading. The spelling error has been corrected.

**Changes:**

- Table 3

p. 11, 209-2020: This paragraph could be very much condensed by simply stating that “many of the symptoms of MDD that attracted high online interest correspond to those included in the DSM-
5-TR diagnostic criteria” and just providing one or two examples for the corresponding symptoms.

**Response:**

Thank you for this helpful comment. We have simplified the explanation, specifically highlighting only sleep disturbances and headaches, while summarizing the remaining symptoms in a single paragraph.

**Changes:**

- As shown in Table 3, many of the high-volume search queries related to depressive symptoms corresponded to the symptoms listed in the DSM‑5‑TR criteria. (Discussion; lines 246 to 248.)
- [Deleted] ~~Feeling/behaving irritable/angry also ranked within the top four symptoms with the highest search volumes in all 3 years. Irritability or uncreased reactivity is reported to be present in approximately 40% of individuals with MDD [21, 22]. However, these symptoms are also reportedly present in 56–67% of individuals with anxiety disorders, including generalized anxiety disorder, obsessive-compulsive disorder, panic disorder, post-traumatic stress disorder and social anxiety disorder [22]. Therefore, those searching for feeling/behaving irritable/angry may have included not only individuals with MDD but also individuals, or their associates, with anxiety disorders.
  General weakness/tiredness ranked fifth in 2024, sixth in 2023, and seventh in 2022, consistently occupying a high position among symptom searches. General weakness/tiredness is reported to be present in over 40% of individuals with MDD [23]. MDD and fatigue are known to be strongly associated. For example, individuals with either MDD or fatigue are approximately twice as likely to experience both [24]. Furthermore, one study found that 69% of patients with MDD present with somatic symptoms as their chief complaint [25]. Hence, actively considering MDD when patients report general weakness/tiredness may facilitate early diagnosis.
  Memory disturbance was another frequently searched MDD symptom term, ranked fourth in 2022, fifth in 2023, and seventh in 2024. Memory disturbance is present in approximately 30% to 60% of individuals with MDD [26, 27]. In individuals with MDD, memory disturbance is closely associated with reduced social functioning which may manifest as employment problems and negatively affects not only the affected individual but also those around them [28]. Therefore, memory disturbance should not be overlooked in treatment of MDD and requires appropriate and adequate intervention.~~
- Feeling/behaving irritable/angry, weakness/tiredness general, and memory disturbance are frequently searched and have been reported to be present in over 40% of individuals with MDD [29-32]. These symptoms are consistent with the DSM diagnostic criteria, and, similar to sleep disturbance, the findings suggest that symptoms frequently observed in clinical practice also attract considerable attention online. (Discussion; lines 259 to 263.)
- Headache was the query with the highest search volume in 2023 and 2024 among somatic symptoms not listed in the DSM-5-TR diagnostic criteria, excluding those classified as residual symptoms. (Discussion; lines 264 to 266.)

p. 12, 247: There seems to be a spelling mistake in “Irritability or uncreased reactivity”.

**Response:**

In response to the previous comment, we have deleted the sentence that included this mistake.

**Changes:**

- [Deleted] ~~Irritability or uncreased reactivity is reported to be present in approximately 40% of individuals with MDD [21, 22].~~

p. 14, 281: The authors should very briefly discuss here who the population subgroups are that do not use the Internet in Japan, for instance, older individuals.

**Response:**

We have specified older adults as an example of the population in Japan that does not use the Internet.

**Changes:**

- Second, owing to the study design, the results do not reflect the level of interest in MDD among population subgroups that do not use the internet such as older adults. (Discussion; lines 283 to 284.)

p. 14, 286 – 291: The authors nicely discuss several reasons why also individuals that do not suffer from MDD might nevertheless search for MDD. To strengthen their discussion of this limitation, they should add that increased public awareness and media attention to MDD and specific MDD symptoms might also lead to more people searching for the topic, simply out of interest without any personal connection to the issue. A good example for this issue is also Google Flu Trends (e.g., Lazer et al. 2014) which was a service provided by Google until 2015 to predict influenza-like illnesses by using the Google search volume for flu symptoms. However, Google Flu Trends largely overestimated influenza outbreaks compared to official data, in parts, because individuals that did not have the flu also googled flu symptoms.

**Response:**

We appreciate the reviewer for pointing out this important concern. We have included increased media coverage as a factor that may lead individuals without MDD to search for MDD.

**Changes:**

- In addition, increased media coverage of celebrities or health-related information about depression may lead to higher search volumes driven purely by public interest. (Discussion; lines 293 to 295.)

## **Others**

We also indicated other minor corrections in yellow markers (please see “Revised Manuscript with Track Changes”).

We thank the reviewer for such pertinent comments. We hope that the revised manuscript contains suitable responses to the comments and has been significantly improved compared to the previous submission. We trust that our manuscript is now suitable for publication in PLOS ONE.
