# Supplementary material for: Online search interest in major depressive disorder: Infodemiology study using the most visited search engine in Japan
Source: PLoS One. 2026 May 12;21(5):e0349091. doi: 10.1371/journal.pone.0349091 (PMC13166940; doi:10.1371/journal.pone.0349091)
Supplement: Attachment — Submitted filename: Response-to-Reviewer_Shimizu_auresp_2.docx [file pone.0349091.s004.docx]

April 16, 2026

Dear Dr. Akaishi,

**Title:** Online Search Interest in Major Depressive Disorder: Infodemiology Study Using the Most Visited Search Engine in Japan

**Reference number:** PONE-D-25-64954R1

Dear Dr. Akaishi,

Thank you for your e-mail of April 6, 2026, regarding our manuscript, “**Online Search Interest in Major Depressive Disorder: Infodemiology Study Using the Most Visited Search Engine in Japan**”, and for the valuable comments of the reviewer. I have attached our revised manuscript, as well as a point-by-point response to the reviewer’s comments.

We hope that the revised manuscript contains suitable responses to the comments, and we think that it has been significantly improved over the previous submission. We trust that our manuscript is now suitable for publication in PLOS ONE.

Thank you in advance for your kind consideration of our work.

Sincerely yours,

Kosuke Ishizuka, MD, PhD

Department of General Medicine, Yokohama City University School of Medicine

3-9 Fukuura, Kanazawa-ku, Yokohama-city, Kanagawa pref. Japan

Tel. +81-45-787-2800

Fax. +81-45-350-2728

E-Mail: e103007c@yokohama-cu.ac.jp

**RESPONSES TO REVIEWER 2:**

We sincerely appreciate the reviewer’s thoughtful comments, which have greatly contributed to improving our manuscript. We believe that the revised version has adequately addressed all the points raised and represents a substantial improvement over the previous submission. We hope that the manuscript is now suitable for publication in PLOS ONE.

## **NOTE**

We highlighted changes of significant issues in yellow (please see “Revised Manuscript with Track Changes”).

**Comments:**

I thank the authors for considering my earlier comments in their revision of the manuscript “Online Search Interest in Major Depressive Disorder: Infodemiology Study Using the Most Visited Search Engine in Japan”. I especially appreciate the careful revision of the presentation and discussion of the results. I see most points implemented very well which leaves me with only minor remarks that I outline in detail below. I hope my comments are constructive and helpful to the authors in further strengthening their manuscript.

**Response:**

We appreciate your overall comments. The manuscript has been revised in line with your suggestions, and all issues raised have been carefully addressed.

**Major remarks:**

1. Relevance and goal (e.g., lines 34ff. and 50f. in the abstract, line 80f. in the introduction, line 325f. in the conclusion):
I highly appreciate the changes made in the manuscript further clarifying the study’s goal and relevance. However, I still find it difficult to understand how diagnostic practices and the measurement of public interest in MDD are conceptually linked. Specifically, it remains unclear to me how societal perceptions translate into or influence clinical diagnostic practices. I am still missing an explicit explanation of this intermediate step.
In the conclusion, the authors state that “This line of research may help primary care physicians to proactively identify patients with MDD by focusing on these commonly searched symptoms” (line 325f.). This statement makes the connection most clear compared to the abstract and introduction. However, what does identify mean in this context? Does this imply that practitioners should more proactively ask about the most searched symptoms? If so, the study’s implications are less about improving our understanding of diagnostic practices but may instead improve diagnostic practices.
At the same time, I am still wondering whether practitioners are not already asking about these symptoms anyways, given that they are listed in the clinical diagnostic catalogue. What are currently the reasons for the underdiagnosis mentioned in line 70f. and how could knowing search interest in specific symptoms help to overcome them?
Additionally, it remains unclear how the study contributes to early diagnosis if individuals searching for these terms may never consult a physician who could diagnose MDD. This gap in the argument should also be addressed in the manuscript.
Put differently, the discussion section would benefit from a clearer explanation of what practitioners can concretely learn from the finding that “public awareness of depressive symptoms may be partially aligned with clinical diagnostic concepts” (line 250f.).

**Response:**

We thank the reviewer for this valuable comment. After careful consideration, we have revised the study objective to make it more appropriate. We have also added an explanation in the Introduction regarding the fact that depression may remain undiagnosed even among patients who seek medical care. Furthermore, we have clarified in the Abstract, Discussion, and Conclusion how societal perceptions could contribute to identifying patients who have already accessed healthcare services but remain undiagnosed.

**Changes:**

- This study aimed to describe online search trends on MDD in Japan and investigate the relationship between societal perceptions of depression and the symptoms constructs in clinical diagnosis. (Abstract; lines 34 to 36.)
- Keeping in mind symptoms that attracted high attention online during clinical interviews may contribute to more proactive detection of MDD in primary care settings. (Abstract; lines 50 to 52.)
- In a multinational study of primary care facilities across 15 countries, 45% to 95% of patients with MDD presented with somatic symptoms [10]. Among patients with MDD and anxiety disorders, physicians recognized 77% of those who presented with psychosocial symptoms, but only 22% of those who presented with somatic symptoms alone. [11]. (Introduction; lines 73 to 77.)
- Additionally, we sought to investigate the relationship between societal perceptions of depression and the symptoms constructs in clinical diagnosis. (Introduction; lines 86 to 88.)
- Being mindful that not only commonly reported physical symptoms but also symptoms that attracted high levels of attention online are likely to be widely recognized by the public could help primary care physicians conduct more effective clinical assessments and improve the identification of MDD. Future research should investigate the relationship between the presence of depression or help-seeking behaviors and search activity to further strengthen these interpretations. (Discussion; 283 to 288.)
- Keeping in mind symptoms that attracted high attention online during clinical interviews may contribute to more proactive detection of MDD in primary care settings. Further research is needed to determine the extent to which such behavior reflects clinical condition. (Conclusion; 335 to 338.)

2. On the manual query classification of symptoms:
I also very much appreciate the clarifications to the manual coding process of the queries. However, I still have several questions and uncertainties regarding the coding process:
• I fully understand that the data cannot be shared due to the policies of Yahoo! JAPAN Corporation. I do, however, think that the authors need to share a codebook in the Appendix showing the coding rules for each symptom and a (synthetic) query example when a category was coded. Even if the data cannot be shared, readers need to be able to understand when a query was coded as a specific symptom.
• What are the representative classification rules the authors refer to in “Representative classification rules were applied” (line 136f.)? Providing the codebook would also help here in understanding based on which coding rules the queries were coded. The authors should also state how they developed the classification rules (i.e., iteratively and deductively).
• How did the authors choose the 93 queries that were double coded by two researchers? I assume that they have not been randomly selected as they are all from the year 2024 (which is also why I would not use the term representative in this context as in “representative example” in line 139).

**Response:**

Thank you for your important comment. We have added a codebook for the classification of symptom-related search queries based on ICPC-2 as S1 Table. Accordingly, we revised the Methods. In addition. We clarified that the 93 symptom queries from 2024 used to calculate Cohen’s kappa represent all symptom queries for that year.

**Changes:**

- For example, both “Depression can’t sleep” and “Depression just sleeping all day” were classified under “Sleep disturbance”, allowing for consistent categorization across slang and variant expressions. (Methods; lines 143 to 145.)
- As a representative example, Cohen’s kappa coefficient was calculated for all 93 symptom queries from 2024, yielding κ = 0.751, indicating substantial inter-rater agreement and sufficient reproducibility of the classification. (Methods; lines 145 to 148.)
- A detailed codebook for the classification of search queries into ICPC-2 categories is provided in S1 Table. (Methods; lines 148 to 149.)

**Minor remarks:**

1. Line 120: It would be great to add here whether the “Communications Usage Trend Survey” is a probabilistic or non-probabilistic survey.

**Response:**

Along with your comments, we have added a statement that the “Communications Usage Trend Survey” is a probabilistic survey.

**Changes:**

- The “Communications Usage Trend Survey” is a probabilistic survey conducted to assess trends in telecommunications usage, included 39,577 respondents in 2022, 34,196 in 2023, and 37,058 in 2024 [20-22]. (Methods; lines 126 to 129.)

2. Line 115: In “The internet user volume was defined as the number of internet users in Japan”, it might make the definition even more clear to specify “the absolute number of internet users in Japan”.

**Response:**

In response to the reviewer’s comment, we revised the Methods.

**Changes:**

- The internet user volume was defined as the absolute number of internet users in Japan. (Methods; line 122.)

3. Line 197: The dot in “Depression test.” should come after the quotation mark.

**Response:**

We appreciate the reviewer’s careful reading. We have corrected the typographical error.

**Changes:**

- The second and third most common queries in each year were “Depression” or “Depression test”. (Results; lines 204 to 205.)

We appreciate the reviewer for such helpful comments. We believe that the revised manuscript has been improved through these revisions and that the reviewer’s concerns have been addressed. We hope that the revised version is now suitable for publication in PLOS ONE.
